# Evaluation of the modified Pittsburgh classification for predicting the disease‐free survival outcome of squamous cell carcinoma of the external auditory canal

**DOI:** 10.1002/hed.26424

**Published:** 2020-08-13

**Authors:** Cindy H. Nabuurs, Wietske Kievit, Nilou Labbé, C. René Leemans, Conrad F. G. M. Smit, Michiel W. M. van den Brekel, Robert J. Pauw, Bernard F. A. M. van der Laan, Jeroen C. Jansen, Martin Lacko, Weibel W. Braunius, Shinya Morita, Małgorzata Wierzbicka, Takuma Matoba, Nobuhiro Hanai, Robert P. Takes, Henricus P. M. Kunst

**Affiliations:** ^1^ Department of Otorhinolaryngology and Head and Neck Surgery Radboud University Medical Center Nijmegen The Netherlands; ^2^ Rare Cancers Radboud Institute for Health Sciences Nijmegen The Netherlands; ^3^ Department of Health Evidence Radboud University Medical Center Nijmegen The Netherlands; ^4^ Department of Otolaryngology—Head and Neck Surgery Amsterdam University Medical Centers, VU University Amsterdam The Netherlands; ^5^ Department of Head and Neck Surgery Netherlands Cancer Institute—Antoni van Leeuwenhoek Amsterdam The Netherlands; ^6^ Department of Otorhinolaryngology and Head and Neck Surgery Erasmus Medical Center Rotterdam The Netherlands; ^7^ Department of Otorhinolaryngology and Head and Neck Surgery University of Groningen, University Medical Center Groningen Groningen The Netherlands; ^8^ Department of Otorhinolaryngology and Head and Neck Surgery Leiden University Medical Center Leiden The Netherlands; ^9^ Department of Otorhinolaryngology and Head and Neck Surgery Maastricht University Medical Center Maastricht The Netherlands; ^10^ Department of Head and Neck Surgical Oncology University Medical Center Utrecht Cancer Center Utrecht The Netherlands; ^11^ Department of Otolaryngology–Head and Neck Surgery Hokkaido University Graduate School of Medicine Sapporo Japan; ^12^ ENT Department Medical University Pozna'n Poland; ^13^ Department of Otorhinolaryngology and Head and Neck Surgery Nagoya City University Hospital Nagoya Aichi Japan; ^14^ Department of Head and Neck Surgery Aichi Cancer Center Hospital Nagoya Aichi Japan

**Keywords:** disease‐free survival, neoplasm staging, prognosis, squamous cell carcinoma, temporal bone

## Abstract

**Background:**

Squamous cell carcinoma (SCC) of the external auditory canal (EAC) is a rare disease, which is commonly classified with the modified Pittsburgh classification. Our aim was to evaluate the predictive performance of this classification in relation to disease‐free survival (DFS).

**Methods:**

We examined retrospective data from a nationwide Dutch cohort study including patients with primary EAC SCC. These data were combined with individual patient data from the literature. Using the combined data, the predictive performances were calculated using the c‐index.

**Results:**

A total of 381 patients were included, 294 for clinical and 281 for the pathological classification analyses. The c‐indices of the clinical and the pathological modified Pittsburgh classification predicting DFS were 0.725 (0.668‐0.782) and 0.729 (0.672‐0.786), respectively.

**Conclusion:**

The predictive performance of the modified Pittsburgh classification system as such appears to be acceptable to predict the DFS of EAC SCC. Other factors need to be added to a future model to improve the predicted performance.

## 1 INTRODUCTION

Primary carcinoma of the external auditory canal (EAC) is a rare disease with an estimated incidence of 1 to 5 cases per million people per year.^1^, ^2^, ^3^ Among these tumors, squamous cell carcinoma (SCC) is the most common histologic type.^1^, ^2^, ^4^ EAC SCC can be extensively invasive to the temporal bone, where critical anatomical structures are located. Because it is such a locally aggressive disease that may metastasize, it is associated with a poor prognosis and a high recurrence rate.^4^, ^5^ The 5‐year disease‐free survival (DFS) of EAC SCC is 29% to 83%, depending on the tumor classification.^6^, ^7^, ^8^, ^9^

The treatment options for EAC SCC consist of surgery, radiotherapy, chemotherapy, or combinations of these. The choice of treatment depends on the classification of the tumor. However, the published studies provide various conclusions on the best treatment and are of limited value due to the small study sample and the use of different tumor classification systems. An adequate and uniform tumor classification system is not only essential to evaluate the various treatments, but also to use it as a support tool for deciding on the optimal treatment and predicting the prognosis of these patients.

Although there is an international standardized staging system for EAC SCC, which has been accepted by the American Joint Committee on Cancer (AJCC),^10^ the modified Pittsburgh classification is currently one of the most commonly used tumor classifications for EAC SCC.^11^, ^12^, ^13^, ^14^ Both classification systems include four T‐classifications (T1‐T4). However, the AJCC classification is mainly based on the diameter of the tumor and the modified Pittsburgh classification on the erosion of anatomical structures by the tumor. Several studies have shown that the modified Pittsburgh classification distinguishes in overall survival (OS) between the four T‐classifications.^8^, ^11^, ^13^, ^15^, ^16^, ^17^, ^18^, ^19^ As (local) recurrent or residual disease can have a serious impact on the patients quality of life, DFS should be considered a more clinically relevant outcome measurement for EAC SCC. However, the predictive potential of the Pittsburgh classification to predict the DFS of individual patients with EAC SCC has not yet been evaluated in a large study sample. This is crucial before implementing a classification system as predictive tool in clinical practice.

The primary aim of this retrospective study is to evaluate the predictive performance of the modified Pittsburgh classification by comparing the DFS outcome of each T‐classification, using Kaplan‐Meier analyses. The predictive performance of the modified Pittsburgh classification was also analyzed in a multivariable Cox regression model, including the T‐classification and nodal status as predictors. To pursue this aim, we established a Dutch nationwide database, reviewed the literature systematically and combined our nationwide database with the individual patient data of existing retrospective studies for statistical analyses.

## 2 METHODS AND MATERIALS

### 2.1 Data collection for the national data

A nationwide cohort study was conducted on patients who were treated curatively for their primary EAC SCC in one of the eight Dutch head and neck oncological centers (Radboud University Medical Center; Amsterdam University Medical Center; Netherlands Cancer Institute—Antoni van Leeuwenhoek; Erasmus Medical Center; University Medical Center Groningen; Leiden University Medical Center; Maastricht University Medical Center; University Medical Center Utrecht). Two nationwide coding systems (ICD‐code, “International Statistical Classification of Diseases and Related Health Problems” and PALGA, “Pathologisch‐Anatomisch Landelijk Geautomatiseerd Archief”) were used to obtain data of patients who were diagnosed with SCC of the EAC, temporal bone or middle ear. Thereafter, the diagnosis was verified manually by checking the imaging and pathological results carefully by CN. Patients were excluded if they received palliative care for their primary EAC SCC; the site of the origin was not the EAC, temporal bone or middle ear; the histologic subtype was not SCC; or if the medical records were too limited to classify the tumor. Patients were also excluded if they were treated by local resection with local application of 5‐fluorouracil or if they did not receive surgery, in order to improve the homogeneity of the data. In order to increase the database on this rare subject as much as possible and assuming that the improved treatment techniques over the years do not significantly affect the outcome, no inclusion or exclusion criteria were applied to specified dates of diagnosis.

From the patients included, the data were obtained retrospectively. All clinical and pathological staging was done according to the modified Pittsburgh classification. ^11^ The clinical TNM‐classification was based on clinical exam reports and imaging results and the pathological T‐ and N‐classifications were adjusted if necessary based on the operative reports and pathological results of the temporal bone resection specimens.

Approval was obtained from the medical ethics committee of Radboud University Medical Center (number 2017‐3397); participating centers complied with their local medical ethics committee requirements.

### 2.2 Data collection for the individual patient data from the existing literature

To complement our cohort, we also obtained individual patient data of existing studies identified by a systematic review of the literature. Details of the protocol of this systematic review were registered on PROSPERO (registration number: CRD42017073053) and it can be accessed at http://www.crd.york.ac.uk/PROSPERO/display_record.php?ID=CRD42017073053.

A systematic literature search was conducted between 24 July 2017 and 31 August 2017 in PubMed, EMBASE, and Cochrane Library using the terms and the synonyms of “EAC,” “temporal bone,” “middle ear,” “SCC,” and “survival.” Two authors, CN and NL, independently screened the title and abstract of the retrieved articles to identify studies meeting the inclusion criteria. Thereafter, full texts of the remaining articles were screened by CN and NL. Discrepancies were solved by discussion and consensus. We included studies of patients with primary SCC of the EAC, temporal bone or middle ear. The other inclusion criteria were that the studies also have reported the tumor classification or the required information in order to classify, according to the modified Pittsburgh classification, treatment strategy, type of resection, and the occurrence of and time to a recurrence. Studies that were not clear on which patients had primary EAC SCC, that were not clear whether the TNM classification was based on clinical results or adjusted on pathological results, that did not present clear outcomes for individual patients, or that were written in languages other than Dutch or English, were excluded. In order to ensure that the data reported in the literature did not overlap with data from the Dutch nationwide cohort, we excluded studies including patients treated in a Dutch center. There were no inclusion or exclusion criteria on specified dates of publication or dates of treatment in order to gain a sample as large as possible. Patients were excluded if they did not receive surgery or were treated with palliative intent in order to improve the homogeneity of the data.

Some authors presented data of individual patients in their paper itself. If this was incomplete or the study did not provide the individual patient data, the authors were contacted by electronic mail to ask for their raw data. If the authors did not respond directly or after reminders resulting in incomplete individual patient data, the study concerned was excluded. If a part of the study population met the inclusion criteria, only the individual patient's data of these subjects were extracted.

### 2.3 Statistical analysis

The predicting performance of the modified Pittsburgh classification was examined in the combined sample, including patients in the Dutch nationwide database and those obtained from previously published retrospective studies. Database consisted of the following variables: gender, age, facial nerve paralyses, cT‐classification, pT‐classification, cN‐classification, pN‐classification, clinical suspected lymph node metastasis, pathological proven lymph node metastasis, treatment strategy, surgical treatment, surgical technique, parotidectomy, neck dissection, radiotherapy, chemotherapy, surgical margins, outcome, recurrence/residual, DFS, DFS outcome in months, OS, OS outcome in months, and follow up.

All analyses were performed separately for the clinical and pathological TNM classification. Most of the studies obtained from the systematic literature search presented either only the clinical or the pathological tumor classification, but not both classifications. The individual patient data containing only the clinical classification were only used for the statistical analysis of the clinical classification. The individual patient data containing only the pathological classification were only used for the statistical analysis of the pathological classification.

In order to evaluate the predictive performance of the modified Pittsburgh classification consisting of the T‐, N‐ and M‐classifications, a Cox proportional hazard model was fitted with these classifications as fixed covariates and DFS as primary outcome. DFS was defined as the time in months from the start of treatment for EAC SCC until recurrence. The DFS of residual diseases were considered as zero months. Patients were censored at the moment of their last visit, when they died not due to EAC SCC or when they were lost to follow‐up. Cox proportional hazard models were also created with overall survival as outcome. OS was defined as the time in months from the start of treatment for EAC SCC until death caused by any reason. Patients were censored at the time of their last visit or when they were lost to follow‐up. However, the subgroups per N‐classification appeared to be too small to analyze, so the presence or absence of lymph node metastasis (N0 vs N+, respectively) as such was used for statistical analyses instead of separate N‐classifications. Because only patients treated with curative intent were included for this study, every tumor included was classified as a clinical and pathological M0. As a consequence, this variable was excluded as covariate in our statistical analysis.

First, the raw data were explored by graphically presenting the DFS per T‐classification and the presence of lymph node metastasis using the Kaplan‐Meier survival curves. The differences between the 5‐year DFS outcome across the T‐classifications were analyzed using the log‐rank test. The log‐rank test was also used to analyze the difference between the 5‐year DFS outcome between the presence and absence of lymph node metastasis. These analyses were performed using the available raw data.

Thereafter, missing data were imputed by multiple imputation in order to prevent inefficient analyses and biased estimates of the associations investigated using Cox regression analyses. The multiple imputation was performed using the “mice” package (version 2.46.0) in the R software version 3.4.3 (R Project for Statistical Computing).^20^ This method assumes that data are missing at random. Data were missing in 16 of the 24 variables (range of 2.38%‐36.39%) in the dataset containing only subjects who had at least the clinical tumor classification. In the dataset containing only subjects who had at least the pathological tumor classification, data were missing in 14 of the 24 variables (range of 0.36%‐30.96%). Variables with more than 15% of missing data were the pathological T‐classification and pathological lymph node metastasis for the database including patients with clinical classification. For the database including patients with pathological classification database, these variables were the clinical T‐classification and clinical suspected lymph node metastasis. Separate multiple imputations were performed for the clinical and for the pathological classification data, but using the same technique: we used all variables (including information about the treatment, baseline variables and outcomes) to predict the missing values in 10 imputed datasets based on 10 iterations and 10 multiple imputations. The pooled multiple imputed data were used to develop mixed effect Cox proportional hazard regressions including individual T‐classifications (as fixed covariate), the presence of lymph node metastasis (as fixed covariate), and the studyID (included centers and studies, as random effect) on the 5‐year DFS and 5‐year OS.

To evaluate the predictive performance of our fitted model the concordance index (c‐index) was calculated as is common in the field of prediction modeling. The imputed data were used to calculate this c‐index. A c‐index >0.70 was considered as a sufficient predictive performance and a c‐index >0.80 as an adequate predictive performance.^21^

All statistical analyses were performed in R version 3.4.3 (R Project for Statistical Computing).^20^ In all analyses, a probability (*P*) value <.05 was considered statistically significant.

## 3 RESULTS

### 3.1 Results from national data

In total, 289 patients were diagnosed with primary EAC SCC between 1975 and 2017. Of these patients, 103 patients were excluded because they were not treated with curative intent (n = 68), were treated by local resection with local application of 5‐fluorouracil (n = 25), or did not receive surgery (n = 10). Finally, 186 patients were included for this study. The mean age was 65.9 years; 48.4% of the study population was male and 51.6% was female; 39.2% of the tumors was classified as a pT4; 15.6% had pathologically proven lymph node metastasis; 58 T‐classifications were adjusted based on operative reports and pathology results; the median follow‐up was 34 months with a minimum of 1 month and maximum of 344 months; and 68 patients had residual disease or recurrence. Table 1 shows the patient's characteristics per medical center.

**TABLE 1:** Patient characteristics for the Dutch national cohort, by medical center

Dutch medical center	Number of patients	Median age in years and (range)	Number of male (%)	pT1 (n)	pT2 (n)	pT3 (n)	pT4 (n)	Presence of lymph node metastasis (%)	Median FU in months (range)
1	52	68.5 (38‐91)	20 (38.5)	19	4	7	22	5 (9.6)	35 (1‐344)
2	40	69 (29‐90)	21 (52.5)	8	6	13	13	9 (22.5)	28 (3‐267)
3	10	67.5 (37‐81)	5 (50)	4	2	2	2	4 (40)	51 (8‐344)
4	9	68 (40‐77)	8 (88.9)	1	1	3	4	2 (22.2)	29 (8‐62)
5	18	63.5 (39‐78)	8 (44.4)	3	2	4	9	3 (16.7)	39 (4‐235)
6	24	64.5 (49‐89)	15 (62.5)	9	2	2	11	3 (12.5)	56 (1‐209)
7	10	72.5 (49‐85)	4 (40)	1	0	6	3	2 (20)	13 (4‐116)
8	23	68 (49‐84)	9 (39.1)	7	2	5	9	1 (4.3)	41 (4‐122)
Total	186	67 (29‐91)	90 (48.4)	52 (28%)	19 (10%)	42 (23%)	73 (39%)	29 (15.6)	34 (1‐344)

Abbreviations: FU, follow‐up.

### 3.2 Results from literature study and using the individual patient data from the existing literature

**FIGURE 1:**
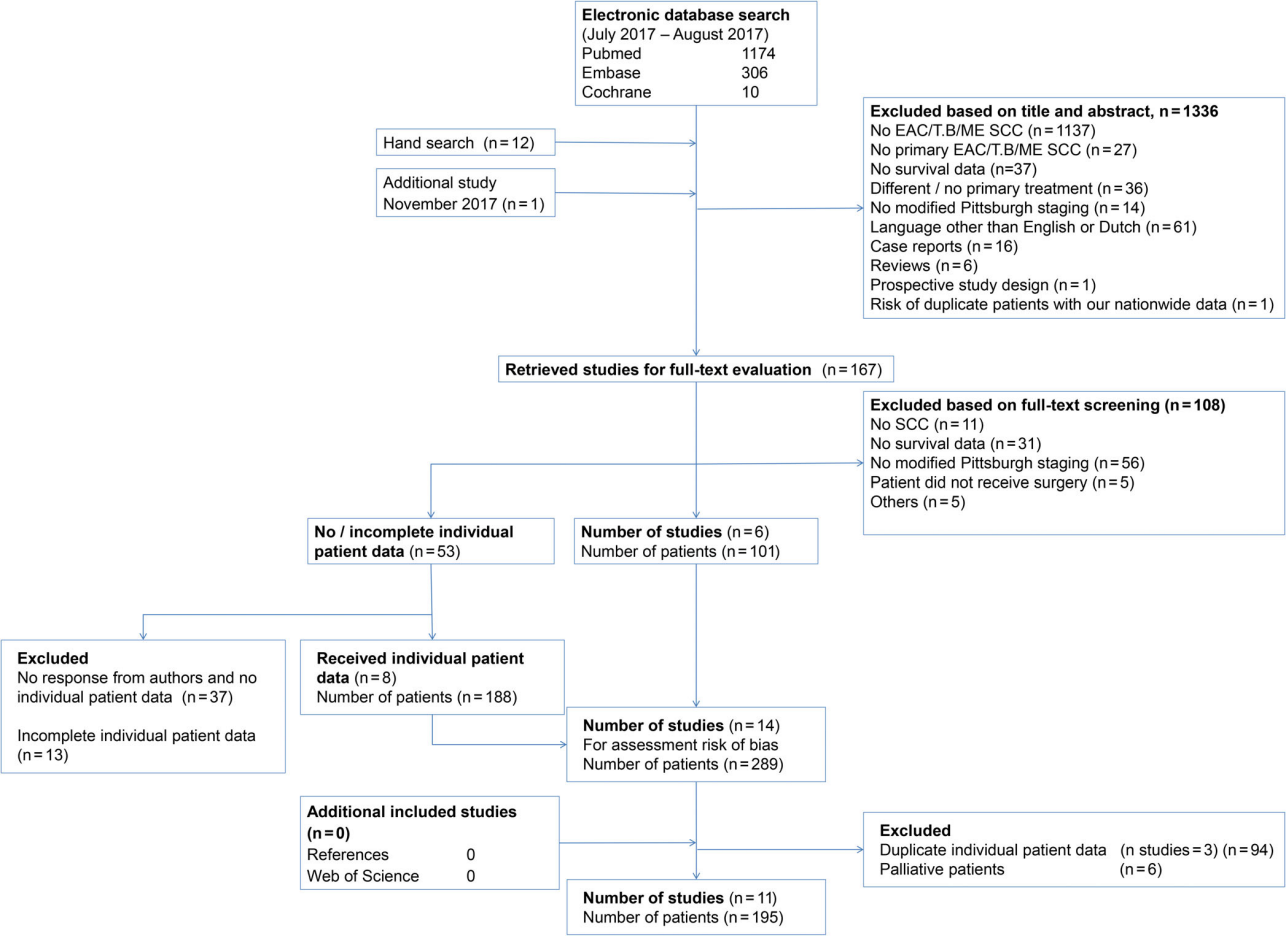
Flowchart of studies [Color figure can be viewed at wileyonlinelibrary.com]

Our search strategy identified 1490 studies. Figure 1 shows the flowchart of the inclusion and exclusion of studies. After applying these inclusion and exclusion criteria, 11 studies were included with a total of 432 patients. Table 2 provides general information of the articles included. Of the 432 patients, 195 patients met the inclusion criteria and remained for further analyses. Of the patients included 44 patients experienced residual disease or recurrence. The characteristics of the studies included are provided in Table 3. All studies included were retrospective studies published between 2000 and 2017. Patients of the studies included were treated between 1978 and 2014. Although the follow‐up of the studies were in general relatively short with a median of 34.8 months (1‐211 months) and not all studies described both the clinical and pathological tumor classifications (2 of 11 studies), the individual patient's data of the studies included contained the necessary data for our statistical analyses.

**TABLE 2:** Characteristics of studies included

Reference	Year of inclusion	Number of patients mentioned in the published studies	Number of patients included for this study	Reason of exclusion
Choi et al^22^	August 1989 to March 1996	21	11	11 patients were diagnosed with adenoid cell carcinoma
Kawahara et al^23^	1994 to 2006	17	7	2 recurrences; 8 patients were diagnosed with other histologic types of tumors
Lassig et al^13^	January 1995 to April 2007	30	13	14 patients with tumors of auricle/preauricular/parotid/or postauricular; 3 tumors were unresectable and we assumed that these patients were not treated with curative intent
Matoba et al^24^	January 1999 to December 2014	25	10	3 patients with auricle tumor; 4 patients had other histologic type; 1 tumor was a recurrence; 7 patients were treated without surgery
Moody et al^11^	1978 to 1998	32	32	
Moore et al^25^	1990 to 2007	35	17	The published article included 15 patients with tumors consisting of other histologic type than SCC; we received data of 21 patients with SCC^a^ and we excluded 3 patients because these tumors were located at auricle; 1 patient because the tumor was a recurrence of a parotid tumor
Morita et al^16^	April 1997 to March 2015	66	34	32 patients did not receive surgery
Morris et al^26^	1994 to 2010	72	19	We received data of 81 patients^a^ of which 61 patients were excluded because of the following: 41 patients with other tumor site than middle ear, external auditory canal, or temporal bone; 14 patients with other histologic type; 6 patients who were treated previously for a tumor in the same region; and in 1 patient radiotherapy was stopped early due to progression of the disease along skull base
Nakagawa et al^27^	January 1998 to March 2004	25	11	1 patient had previous radiotherapy at the tumor location; 2 patients were treated with palliative intent; 11 patients had no surgery
Nyrop and Grøntved^28^	January 1979 to July 2000	20	10	10 patients had tumors with other histologic type than SCC
Wierzbicka et al^29^	January 2006 to December 2010	89	31	We received data of 92 patients^a^; 21 patients had tumors at other locations; 21 patients had other histologic type than SCC; 19 patients were treated previously for a tumor in the same region

Abbreviations: SCC, squamous cell carcinoma.

^a^ The author sent us data from the patients included in their published studies as well as data from patients who were included in their database after the studies were published.

**TABLE 3:** Characteristics of patients included from the literature

Source	Number of patients^a^	Median age in year (range)	Number of male (%)	Clinical (C) or pathological (P) classification	T1	T2	T3	T4	Presence of lymph node metastasis (%)	Median FU in months (range)
Choi et al^22^	11	60 (35‐67)	7 (63.6)	C	2	3	2	4	0 (0)	29 (6‐136)
Kawahara et al^23^	7	62 (41‐69)	4 (57.1)	C	0	0	1	6	NR	62.4 (8‐88)
Lassig et al^13^	13	70 (31‐88)	7 (53.8)	P	4	3	2	4	4 (30.8)	48 (1‐150)
Matoba et al^24^	10	62.5 (57‐76)	5 (50)	P	3	2	2	3	3 (30)	33.5 (10‐196)
Moody et al^11^	32	72 (42‐92)	NR	P	7	5	6	14	4 (12.5)	20 (5‐211)
Moore et al^25^	17	62 (27‐83)	8 (47.1)	P	6	1	4	6	3 (17.6)	49 (5‐173)
Morita et al^16^	34	65 (29‐86)	17 (50)	C	13	9	8	4	5 (14.7)	37.5 (4‐112)
Morris et al^26^	19	64 (32‐85)	13 (68.4)	C	4	0	3	2	1 (5.3)	29 (6‐188)
				P	9	0	3	7	2 (10.5)	
Nakagawa et al^27^	11	61 (46‐80)	6 (54.5)	C	0	1	3	3	1 (14.3)	48 (4‐84)
				P	1	2	0	1	0 (0)	
Nyrop and Grøntved^28^	10	NR	NR	C	6	0	1	3	0 (0)	32 (6‐141)
Wierzbicka et al^29^	31	56 (20‐82)	14 (45.2)	C	0	2	4	25	5 (16.1)	32 (4‐137)
Dutch nationwide database	186	67 (29‐91)	90 (48.4)	C	57	18	40	70	23 (12.4)	34.0 (1‐344)
				P	52	19	42	73	29 (15.6)	
Total	381	64.5 (20‐92)	171 (44.9)	C (n = 294)	82 (28%)	33 (11%)	62 (21%)	117 (40%)	35 (11.9)	34.0 (1‐344)
				P (n = 281)	82 (29%)	32 (11%)	59 (21%)	108 (38%)	45 (16)	

^a^ The number of patients from the original studies included in the current analyses. Some individual patient's data within these studies included did not meet the inclusion criteria and were excluded, for example, recurrences, adenoid cystic carcinoma, or basal cell carcinoma. FU, follow‐up. NR, not reported.

### 3.3 Patient characteristics

In total 381 patients, 186 patients from the Dutch nationwide database and 195 patients from the individual patient data of 11 previous studies, with primary EAC SCC were included for this study. Mean age of the combined data was 64.5 year; the gender distribution was 44% female, 45% male, and 11% unreported gender; approximately 29.2%, 11.4%, 21.0%, and 38.4% of the tumors were classified as pT1, pT2, pT3, and pT4 respectively; 16% of the tumors were diagnosed with pN+; the median follow‐up was 34 months with a range of one to 344 months; and 112 patients had residual disease or recurrence (Table 3).

Details of the treatment strategies and surgical techniques are summarized in Table 4. All tumors were mainly treated by surgery in combination with radiotherapy within the Dutch nationwide database tumors. Within the individual patient data from previous studies included the cT1‐ and cT2‐classified tumors were mainly treated by surgery only and cT3‐ and cT4‐classified tumors by surgery in combination with radiotherapy or chemoradiation. The most frequently applied surgical technique was the lateral temporal bone resection (LTBR), but some of the T1‐classified tumors were operated by local resection instead of LTBR. T3‐ and T4‐classified tumors were usually operated by LTBR in combination with a parotidectomy and/or neck dissection.

**TABLE 4:** Details of treatment per T‐classification

	Dutch nationwide data				Individual patient data from existing studies				Total			
	cT1	cT2	cT3	cT4	cT1	cT2	cT3	cT4	cT1	cT2	cT3	cT4
N =	57	18	40	70	25	15	22	47	82	33	62	117
Treatment strategy												
Surgery	13 (23%)	1 (5.6%)	5 (12.5%)	5 (7.1%)	19 (76%)	9 (60%)	3 (13.6%)	7 (14.9%)	32 (39.0%)	10 (30.3%)	8 (12.9%)	12 (10.3%)
Surgery + RT	44 (77%)	17 (94.4%)	34 (85%)	59 (84.3%)	2 (8%)	3 (20%)	13 (59.1%)	30 (63.8%)	46 (56.1%)	20 (60.6%)	47 (75.8%)	89 (76.1%)
Surgery + chemotherapy	0 (0%)	0 (0%)	0 (0%)	0 (0%)	0 (0%)	0 (0%)	0 (0%)	0 (0%)	0 (0%)	0 (0%)	0 (0%)	0 (0%)
Surgery + CRT	0 (0%)	0 (0%)	1 (2.5%)	6 (8.6%)	0 (0%)	3 (20%)	5 (22.7%)	7 (14.9%)	0 (0%)	3 (9.1%)	6 (9.7%)	13 (11.1%)
Missing	0 (0%)	0 (0%)	0 (0%)	0 (0%)	4 (16%)	0 (0%)	1 (4.5%)	3 (6.4%)	4 (4.9%)	0 (0%)	1 (1.6%)	3 (2.6%)
Surgical technique												
Local resection	17 (29.8%)	0 (0%)	5 (12.5%)	6 (8.6%)	8 (32%)	0 (0%)	1 (4.5%)	3 (6.4%)	25 (30.5%)	0 (0%)	6 (9.7%)	9 (7.7%)
LTBR	37 (64.9%)	14 (77.8%)	19 (47.5%)	36 (51.4%)	17 (68%)	13 (86.7%)	12 (54.5%)	20 (42.6%)	54 (65.9%)	27 (81.8%)	31 (50%)	56 (47.9%)
STBR	2 (3.5%)	4 (22.2%)	12 (30%)	24 (34.3%)	0 (0%)	2 (13.3%)	8 (36.4%)	19 (40.4%)	2 (2.4%)	6 (18.2%)	20 (32.3%)	43 (36.8%)
TTBR	1 (1.8%)	0 (0%)	4 (10%)	4 (5.7%)	0 (0%)	0 (0%)	1 (4.5%)	5 (10.6%)	1 (1.2%)	0 (0%)	5 (8.1%)	9 (7.7%)
Missing	0 (0%)	0 (0%)	0 (0%)	0 (0%)	0 (0%)	0 (0%)	0 (0%)	0 (0%)	0 (0%)	0 (0%)	0 (0%)	0 (0%)
Additional surgical resection												
Partial or total parotidectomy	15 (20%)	10 (55.6%)	24 (60%)	46 (65.7%)	2 (8%)	3 (20%)	10 (45.5%)	26 (55.3%)	17 (20.7%)	13 (39.4%)	34 (59.7%)	72 (61.5%)
Neck dissection	10 (13.3%)	8 (44.4%)	16 (40%)	33 (47.1%)	0 (0%)	1 (6.7%)	10 (45.5%)	14 (29.8%)	10 (12.2%)	9 (27.3%)	26 (41.9%)	47 (40.2%)

*Note*: N = 294.

Abbreviations: CRT, chemoradiotherapy; LTBR, lateral temporal bone resection; RT, radiotherapy; STBR, subtotal temporal bone resection; TTBR, total temporal bone resection.

### 3.4 Predictive performance of the modified Pittsburgh classification

**FIGURE 2:**
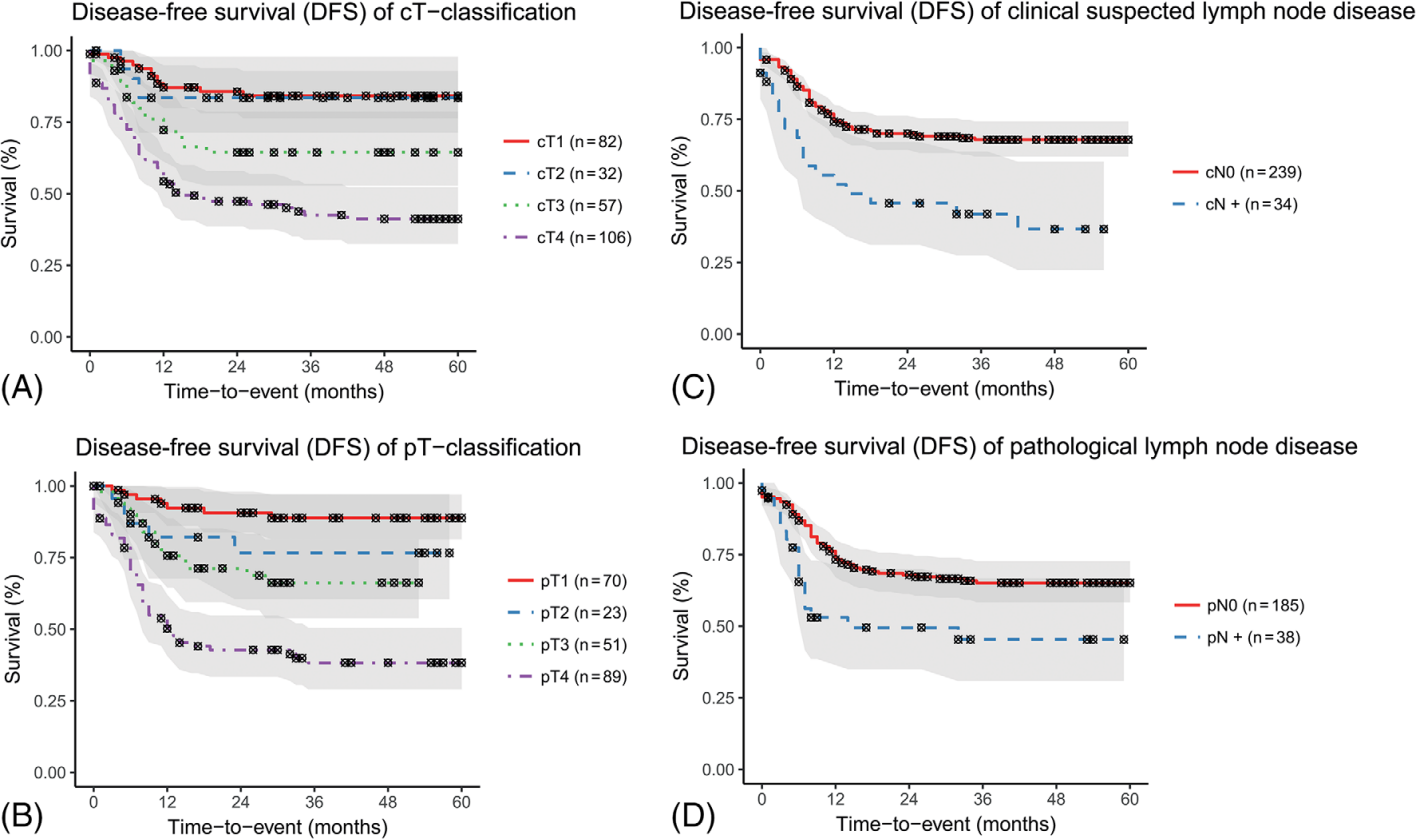
Kaplan‐Meier disease‐free survival curves of the clinical and pathological T‐classification based on the modified Pittsburgh classification and presence of lymph node disease cT, clinical T‐classification; cN0, no clinical suspected lymph node disease; cN+, clinical suspected lymph node disease; pN0, no pathological lymph node disease; pN+, pathological proven lymph node metastasis; pT, pathological T‐classification [Color figure can be viewed at wileyonlinelibrary.com]

As shown in the Kaplan‐Meier survival curves (Figure 2A,B) the 5‐year DFS of cT1‐, cT2‐, cT3‐, and cT4‐classified tumors were 84.2%, 83.5%, 64.5%, and 41.8%, respectively, and of the pT1‐, pT2‐, pT3‐, and pT4‐classified tumors 88.9%, 76.7%, 66.2%, and 39.0%, respectively. However, the log‐rank tests showed that the overall DFS outcomes between T1‐ and T2‐classified tumors (*P* = .84 for clinical classification, *P* = .37 for pathological classification) and between T2‐ and T3‐classified tumors (*P* = .096 for clinical classification, *P* = 0.47 for pathological classification) were not statistically significantly different from each other. The log‐rank tests of the overall DFS outcomes between the remaining T‐classifications were statistically significant (all *P*’s < .05).

The Kaplan‐Meier curves also showed that cN+ had a poorer 5‐year DFS outcome compared with cN0, 36.7% vs 67.9%, respectively. This result was comparable for pN+ compared to pN0, 45.4% vs 65.1% DFS outcome, respectively. The log‐rank tests showed that the DFS outcomes of the cN+ and pN+ were statistically significantly poorer compared to cN0 or pN0, *P* < .001 and *P* < .009, respectively.

The effect of the T‐classifications and presence of lymph node metastasis on the DFS outcome and the OS outcome were analyzed in a mixed‐effects Cox proportional hazard regression in order to calculate the c‐index. The c‐index of the clinical classification to predict the DFS was 0.713 (95% CI = 0.656‐0.770) and of the pathological classification 0.742 (CI = 0.685‐0.799) (Table 5). The c‐index predicting the OS outcome of the clinical classification was 0.668 (CI = 0.609‐0.727) and of the pathological classification 0.734 (0.675‐0.793) (Table 6).

**TABLE 5:** Mixed‐effects Cox proportional hazard regression predicting disease‐free survival

	Hazard ratio	95% confidential interval	*P* value
Clinical modified Pittsburgh classification^a^ (n = 294)			
cT1	Reference		
cT2	1.285	0.452 to 3.649	.64
cT3	2.603	1.250 to 5.422	.011*
cT4	4.798	2.515 to 9.155	<.001***
cN0	Reference		
cN+	1.698	1.016 to 2.838	.044*
*C‐index*	0.713 (0.656‐0.770)		
Pathological modified Pittsburgh classification^b^ (n = 281)			
pT1	Reference		
pT2	1.714	0.564 to 5.208	.340
pT3	1.906	0.828 to 4.386	.130
pT4	4.519	2.106 to 9.695	<.001***
pN0	Reference		
pN+	1.135	0.649 to 1.983	.660
*C‐index*	0.742 (0.685‐0.799)		

Abbreviations: N0, no lymph node metastasis; N+ = lymph node metastasis.

^a^ StudyID as random effect: *χ* ^2^ = 0.74; *P* = .27.

^b^ StudyID as random effect: *χ* ^2^ = 20.04; *P* = .01*.

*Statistically significant (*P* < .05). ***Statistically significant (*P* < .001).

**TABLE 6:** Mixed‐effects Cox proportional hazard regression predicting overall survival

	Hazard ratio	95% confidential interval	*P* value
Clinical modified Pittsburgh classification^a^ (n = 294)			
cT1	Reference		
cT2	0.904	0.364 to 2.249	.830
cT3	1.426	0.779 to 2.609	.250
cT4	2.421	1.431 to 4.094	<.001***
cN0	Reference		
cN+	1.439	0.847 to 2.445	.180
*C‐index*	0.668 (0.609‐0.727)		
Pathological modified Pittsburgh classification^b^ (n = 281)			
pT1	Reference		
pT2	1.533	0.716 to 3.281	.27
pT3	1.548	0.809 to 2.964	.19
pT4	4.006	2.355 to 6.817	<.001***
pN0	Reference		
pN+	1.728	1.084 to 2.754	.021*
*C‐index*	0.734 (0.675‐0.793)		

Abbreviations: N0, no lymph node metastasis; N+, lymph node metastasis.

^a^ StudyID as random effect: *χ* ^2^=3.26; *P* = .28.

^b^ StudyID as random effect: *χ* ^2^=18.80; *P* = .008*.

*Statistically significant (*P* < .05). ***Statistically significant (*P* < .001).

## 4 DISCUSSION

In this retrospective study, we primarily aimed to evaluate the performance of the modified Pittsburgh classification to predict the DFS outcome of patients with primary EAC SCC. It turned out that DFS was statistically significantly different between all T‐classifications except for T2‐ vs T1‐ and T2‐ vs T3‐classified tumor and the predictive performances were acceptably, but with a relatively broad confidential interval of the hazard ratios.

It is difficult to integrate our results with previously described results. First of all, although some studies describe the DFS as clinical outcome, they combined the T1‐ and T2‐classified tumors as early tumors and/or the T3‐ and T4‐classified tumors as advanced tumors.^5^, ^8^, ^19^ In addition, many published studies do not describe the DFS as the primary outcome by default, but rather the overall survival.^11^, ^15^, ^30^, ^31^ For two main reasons, we decided deliberately to analyze primarily the DFS and not the OS. First of all, we think that DFS is clinically more relevant compared to overall survival as outcome, because (local) recurrence or residual disease can have a serious impact on the patients quality of life. Furthermore, OS outcome might be influenced by more confounders such as comorbidities and age compared to DFS outcome. In order to compare our results with other studies, we calculated the OS as secondary outcome. Although the OS outcomes of pT1‐pT4‐classified tumors based on our combined database (2‐year OS of 92%, 74%, 76%, 45%; 5‐year OS of 84%, 66%, 67%, 35%; respectively) are not fully comparable with the studies of Moody et al (2‐year OS of T1‐T4 classified tumors: 100%, 80%, 50%, and 7%, respectively)^11^ and Chi et al (5‐year OS year OS of T1‐T4 classified tumors: 100%, 67%, 21%, and 14%, respectively),^15^ all these results show that the OS outcomes decrease if the T‐classification rises. The differences in OS found in our study compared with these previously published studies might be due in part to the smaller number of participants in the previous studies. However, the predictive performance expressed in a c‐index or area under the curve of the modified Pittsburgh classification itself has not yet been studied, as far as we know. This study shows that the c‐indices for predicting the OS outcome of EAC SCC were about 0.7 (0.67‐0.73), a little less compared to the c‐index for predicting the DFS outcome. This might be explained by the other confounders influencing the OS outcome. If the modified Pittsburgh classification is to be used as a prognostic tool in clinical settings to predict the survival outcome of patients with EAC SCC, an evaluation such as our study is important.

It could be argued whether predictive performances of 0.713 (95% CI = 0.656‐0.770) and of 0.742 (CI = 0.685‐0.799) for DFS are acceptable to use it as a tool in clinical settings or not. An adequate discrimination of a prediction tool can inform clinicians which treatment will give the most optimal survival outcome. However, models with inadequate discrimination performance can have major adverse impacts. The modified Pittsburgh classification was not originally designed as a prediction model: it is used to classify the tumor and to decide the treatment for this cancer as with other tumor classification systems. By inaccurately classifying a tumor too low or too high, there is a risk of under‐ or overtreatment resulting in potentially higher risk of recurrent disease, unnecessary harm, and even death. Our Dutch nationwide database showed that 58 of the 168 clinical T‐classifications were adjusted based on operative reports and pathology results. The clinical T‐classification was based on diagnostic imaging results. However, not all patients received a magnetic resonance scan in addition to the computed tomography, especially not the patients who were diagnosed before the year 2000. Studies showed that magnetic resonance imaging is a valuable diagnostic tool to classify EAC SCC, because it is more sensitive to demarcate the soft tissue extent of the tumor.^32^, ^33^ The adjusted pathological classification may have affected the slight difference between the c‐indices for predicting the DFS of the clinical and pathological classification. Even though this study shows that the modified Pittsburgh classification discriminates acceptably, a higher c‐index may be necessary to use the classification as a predictor.

The limitations in the predictive performance of the modified Pittsburgh classification system as shown in this study might be caused by the T4‐classified tumors. The modified Pittsburgh classification does not discriminate T4‐classified tumors with different directions of tumor invasion (anterior, posterior, medial, lateral, superior, or inferior). Therefore, the treatment and the DFS outcome may differ for tumors within this same T‐classification. Lavieille et al^34^ proposed in 1997 a new subclassification for the T4‐classified tumors, taking into account the direction of the tumor invasion: (T4a) extracranial extension, (T4b) intrapetrous bone and extradural extension, and (T4c) meningeal or intradural involvement.^34^ Zanoletti et al also suggested subclassifying the T4‐classified tumors according to the direction of the invasion of the tumor. Their results showed that tumors spreading anteriorly (parotid space and preauricular region) had a significant higher DFS outcome (87.5%) compared to T4‐classified EAC SCC spreading elsewhere (posterior, superior, inferior, or medial; DFS outcome was 8.3%).^19^ To improve the predictive performance of the modified Pittsburgh classification, a future model might be needed that incorporates a T4‐subclassification.

In addition, the literature already showed that surgical margins are also an important prognostic factor and that positive surgical margins result in significant poorer survival outcome compared to negative surgical margins. The Pittsburgh classification and surgical margins were significant predictors for the prognosis of patients with EAC SCC according to the study of Yin et al.^35^ Morris et al also reported surgical margins as one of the strongest predictors of survival.^26^ Consequently, to optimize the predictive performance for the DFS outcome of patients with EAC SCC, a predictor like surgical margins might need to be added in a future prediction model.

Several limitations to the present study need to be acknowledged. By combining our nationwide database with available individual patient data from prior studies, the sample size for this study was increased and became the largest published cohort study on EAC SCC to our knowledge. Unfortunately, we were unable to include all published studies found in our search. Although we contacted the authors of the concerned studies for additional information, not all the authors could deliver the required data. Collecting prospective data for such a rare disease is not realistic, therefore retrospective data were used. However, one should realize the disadvantages of retrospective data. One of the disadvantages is that 9 of the 11 studies included gave information on clinical or pathological classification only, which made it difficult to combine these data with the nationwide database resulting in two separate databases either including the clinical or pathological classification. When using the individual data of previously published studies, there must be awareness for the risk of false‐positive results by multiple testing. Furthermore, not all individual patient data included information we required, this contributed to missing data up to about 30% for some variables. However, we tried to correct for these missing values by multiple imputation. Even though our combined data resulted in a relatively large database on EAC SCC, the subgroups were still small. The subgroups for N‐classification were even too small for adequate statistical analyses. This is caused by the fact that only 12% to 16% of the patients in this study with EAC SCC had lymph node metastasis. In order to include lymph node metastases in our prediction model, we used the presence and absence of lymph node disease in the Cox regression model instead of separate N‐classifications. Larger databases are needed for adequate statistical analyses on EAC SCC.

The various chosen treatment per T‐classification might also affect the DFS outcome. Because the subgroups per treatment strategy of this retrospective study were too small, the effect of the chosen treatment on the DFS outcome has not been statistically corrected in the Cox regression models. Another limitation of this study is that there is a chance that within the T3 and T4 tumors of the individual patient data derived from prior studies, patients were treated without curative intent and therefore received less aggressive treatments, resulting in worse outcomes compared to similar patients who were treated curatively. Patients who were known to have received palliative treatment were excluded. However, it was not clear from all patients whether they were considered incurable or not. In spite of its limitations, the study certainly adds to our understanding of the predictive performance of the modified Pittsburgh classification.

## 5 CONCLUSION

The predictive performance of the modified Pittsburgh classification system on its own seems acceptable to predict the DFS for patients with EAC SCC. However, we also indicated room for improvement. We suggest to add a revised T4‐classification of the modified Pittsburgh classification and other predicting factors, such as surgical margins, in a future prediction model in order to improve the predicted performance in clinical practice and to use it as a treatment decision support tool for patients with EAC SCC.

## CONFLICT OF INTEREST

The authors declare that there is no conflict of interest.
